# Identifying behaviours for survival and wellness among people who use methamphetamine with opioids in British Columbia: a qualitative study

**DOI:** 10.1186/s12954-022-00630-8

**Published:** 2022-05-19

**Authors:** Jenny Corser, Heather Palis, Mathew Fleury, Jess Lamb, Kurt Lock, Jenny McDougall, Amiti Mehta, Cheri Newman, Heather Spence, Jane A. Buxton

**Affiliations:** 1 BC Centre for Disease Control, 655 W 12th Ave, Vancouver, BC V5Z 4R4 Canada; 2 Faculty of Health Sciences, SFU, Blusson Hall, Room 11300, 8888 University Drive, Burnaby, BC V5A 1S6 Canada; 3 First Nations Health Authority, 1166 Alberni St, Vancouver, BC V6E 1A5 Canada; 4 PEEP- BC Centre for Disease Control, 655 W 12th Ave, Vancouver, BC V5Z 4R4 Canada; 5 KANDU – Kelowna Area Network of Drug Users, Kelowna, Canada; 6 School of Population and Public Health, UBC, 2206 E Mall, Vancouver, BC V6T 1Z3 Canada

**Keywords:** Opioid use, Methamphetamine use, Polysubstance use, Harm reduction, Qualitative study

## Abstract

**Background:**

British Columbia (BC) has been in a state of public health emergency since 2016, due to the unprecedented numbers of fatal and non-fatal drug toxicity (i.e. overdose) events. Methamphetamine detection in illicit drug toxicity deaths increased from 14% in 2012 to 43% in 2020 suggesting a concerning trend of concurrent methamphetamine and opioid use in BC, consistent with rising patterns identified across North America. People who use methamphetamine concurrently with opioids face an elevated risk of harm. This study aimed to identify behaviours for survival and wellness practiced by people who concurrently use methamphetamine and opioids.

**Methods:**

One-on-one semi-structured interviews were conducted by peer research assistants in person and by telephone. Thematic analysis was carried out to identify patterns in behaviours participants described as important to their safety in the context of concurrent use of methamphetamine and opioids.

**Results:**

Participants (*n* = 22) were distributed across the province with at least four participants from each of the five geographic health regions: 64% self-identified as men, and 50% self-identified as Indigenous. Daily methamphetamine use was reported by 72.7% of participants, and 67.3% reported using alone either often or always. Participants made several considerations and adaptations in order to balance the perceived benefits and risks of their use of methamphetamine with opioids. Two overarching themes were identified to describe how participants adapted their use for survival and wellness. The first was personal safety behaviours which included self-regulation and self-care behaviours. The second was interpersonal safety behaviours which included using alongside peers, and engaging with peer-led services (e.g. community outreach organizations) and public health-led services (e.g. overdose prevention sites) to reduce the risk of harm. Participants identified many gaps in available services to meet their diverse needs.

**Conclusions:**

This manuscript identified diversity in participants’ methamphetamine and opioid use (i.e. frequency, route of administration), and a range of behaviours that were performed to improve wellness and survival while using methamphetamine and opioids. Harm reduction and treatment responses must be robust and adaptable to respond to the diversity of patterns of substance use among people who use methamphetamine and opioids concurrently, so as to not perpetuate harm and leave people behind.

## Background

British Columbia (BC), Canada’s third most populous province, has been in a state of public health emergency since 2016, due to the unprecedented numbers of overdoses and overdose-related (illicit drug toxicity) deaths. Despite the introduction and expansion of harm reduction and treatment services in recent years, these numbers have been rising in recent years in BC, driven largely by the presence of fentanyl (a potent opioid) [1]. For example, the death rate has nearly quadrupled since 2015, from 11.1/100,000 to 43.0/100,000 in 2021[1]. Methamphetamine detection in illicit drug toxicity deaths increased from 14% in 2012 to 43% in 2020 [1] suggesting a concerning trend of concurrent methamphetamine and opioid use in BC that reflects rising patterns identified across North America [2–4]. Understanding changes in drug use patterns and perceptions to effectively adapt harm reduction and treatment services accordingly is critical.

Since 2012, a Harm Reduction Client Survey (HRCS) has been administered in BC in order to better understand patterns of drug use and access to services among people who use drugs in the province. Studies conducted using the 2019 HRCS data found significantly higher odds of methamphetamine use among people who use opioids compared to people who did not [5]. The most common self-reported reasons for concurrent use were self-medication, availability and preference, drug effects/properties, and financial or life situation [6]. Although people who use opioids and methamphetamine concurrently are also more likely to own take-home naloxone kits, use observed consumption sites, and prescribe opioid agonist treatment (OAT), risks associated with the illicit drug supply persist [7]. For example, a recent study found that people who used stimulants and opioids concurrently in BC were more likely to experience an opioid overdose compared to people who used opioids alone[7].

Recent international qualitative studies have explored motivations for concurrent methamphetamine and opioid use. They suggest a variety of perceived benefits including: using methamphetamine with opioids prolongs effects of the opioid, using together creates a more desirable experience, and opioid use can reduce the negative effects of ‘comedown’ after methamphetamine use [8]; methamphetamine improves functionality while using opioids [9], polysubstance use is often socially influenced, often used to cope with emotional pain, and impacted by major life events [10].

Despite several perceived benefits outlined in these studies in relation to the concurrent use, there are risks. Although there appears to be a dearth of evidence related to the pharmacological mechanisms of action related to concurrent methamphetamine and opioid use, there is evidence to demonstrate that methamphetamine has a longer half-life than opioids [11]. Theoretically therefore, the timing and dosage of substances could lead to unsafe levels of drug toxicity, increasing risks of overdose, especially when the person using may be unaware of the potency of the substance or drug interactions.

Illicit fentanyl has been identified in more than 80% of opioid samples at drug testing sites in BC and people who use methamphetamine concurrently with opioids face an elevated risk of exposure to harms [12]. In the absence of access to safer alternatives to the illicit drug supply, people who use methamphetamine and opioids must adapt their ways of using to be safer. Our study aimed to identify behaviours that people who concurrently use methamphetamine and opioids practice to be safe. Exploring what people who use drugs do to improve their safety while using drugs is important to better understand perceptions around risk as well as potential misperceptions. These findings can be used to develop health education and interventions to ensure these are implemented in a manner that is relevant and acceptable to people who use methamphetamine with opioids. Ultimately, this will improve the health and wellness of this population.

## Methods

### Study sample and setting

Our study uses quantitative and qualitative data collected as part of the Concurrent Use and Transition to Methamphetamine among people at risk of Overdose (CUT Meth OD) study (CIHR Funding Reference Number 170288). The CUT Meth OD study is a mixed methods study designed to investigate social and systemic factors associated with emergent trends of increased methamphetamine use and use of methamphetamine with other substances. The research team includes people holding academic and or peer research assistant (PRA) roles. PRAs were enrolled from the Professionals for Ethical Engagement of Peers (PEEP) an advisory group of people with lived and living experience (PWLLE) of substance use from across the province who consult on research and evaluation of services involving people who use drugs. PRAs were also enrolled through contacts at other local drug user groups.

Participants were initially recruited through word of mouth from peers and posters at harm reduction sites and drug user advocacy groups. Later in the study, a purposeful sampling approach was used via direct engagement with networks of PWLLE of substance use to increase diversity of representation of the sample by gender, age, ethnicity, and rural/urban residence. Participants represented all five geographic regions of the province, and self- reported using methamphetamine with opioids, were over 16 years of age and spoke English. Following input from PRAs concurrent methamphetamine and opioid use was identified by participants endorsing/self-reporting using methamphetamine with (at the same time as) opioids or using one after the other.

### Data collection

The interview guide was designed in collaboration with PEEP and included questions focused on participant experiences of concurrently using illicit methamphetamine and opioids in the context of an unregulated drug supply.

One-on-one semi-structured interviews were conducted by PRAs in person and by telephone. Telephone interviews have been shown to be methodologically robust for qualitative data collection [13]. Each interview began with demographic data collection and consent. After each interview participants were each provided a $30 cash honorarium for their contributions.

Each interview lasted approximately 30–90 min and were digitally recorded by the PRA. Recordings were labelled with an anonymous identifier and transcribed verbatim. Transcripts were then reviewed by the research team against the recordings for validation and to ensure the removal of personal identifiers. Pseudonyms were subsequently assigned to each participant. Research Ethics approval for this study was received from the University of British Columbia Behavioural Research Ethics Board (REB #: H20-01475).

### Data analysis

Thematic analysis was carried out to identify patterns in the behaviours that participants described as important to their safety in the context of concurrently using methamphetamine and opioids [14]. To gain familiarity with the data, all transcripts were read and reviewed prior to beginning analysis (JC).

Data analysis proceeded as follows: first, all data that reflected safety behaviours were identified and coded by type (e.g. self-regulation, self-care); second, the codes were analysed in search of themes and sub-themes. These themes were defined and summarized with participant quotes and presented to members of PEEP for review to ensure they were presented in a manner that was clear and respectful of the experiences of people who use illicit drugs. Themes and sub-themes were refined according to PEEP’s feedback, which led to a shift in terminology from risk and resilience to survival and wellness. With continued input from members of PEEP, the final presentation of the data was organized into two overarching themes that reflected the ways that people who concurrently use opioids and methamphetamine actively adapt their ways of using for survival and wellness: (1) personal behaviours and (2) interpersonal behaviours.

## Results

### Demographics

A summary of the demographic and drug use characteristics of 22 participants’ who reported using methamphetamine with opioids is shown in Table 1. The participants were distributed across the province with at least four participants from each of the five geographic health regions; 64% self-identified as men, and 50% self-identified as being Indigenous. Participants reported preferred mode of substance use as smoking (45.5%), injecting (40.9%), both smoking and injecting (4.5%), and snorting (9.1%). Daily methamphetamine use was reported by 72.7% of participants, and 67.3% reported using alone either often or always.

**Table 1:** Demographic and substance use profile of participants (*N* = 22)

	*N* (%)/Mean ± SD
*Demographic characteristics*	
Gender	
Man	14 (63.6)
Woman	8 (36.4)
Age, years	40 ± 11.0
Self-reported indigenous ancestry	
First Nations	5 (22.7)
Métis	6 (27.3)
No	11 (50.0)
Health Authority of residence	
Fraser	5 (21.7)
Interior	4 (17.4)
Island	4 (17.4)
Northern	5 (21.7)
Vancouver Coastal	4 (17.4)
Housing	
Private residence	4 (18.2)
Other housing	14 (63.6)
No regular place to stay	4 (18.2)
Employed	
Yes	8 (36.4)
No	14 (63.6)
Substance use characteristics	
Years of illicit drug use	24 ± 10.9
Years of MA use	11 ± 8.1
Preferred route of administration for any substance use	
Smoking	10 (45.5)
Injecting	9 (40.9)
Snorting	2 (9.1)
Smoking and injecting	1 (4.5)
Frequency of MA use	
Daily	16 (72.7)
Often (not defined)	1 (4.5)
Few times a week	3 (13.6)
Few times a month	1 (4.5)
Once a month or less	1 (4.5)
Use alone	
Never	1 (4.5)
Rarely	1 (4.5)
Occasionally	5 (22.7)
Often	9 (40.9)
Always	6 (27.3)
Opioid overdose in last 3 months	
Yes	6 (27.3)
No	16 (72.7)

MA, methamphetamine; Indigenous ancestry included option of First Nations, Métis, Inuit, or no Indigenous ancestry. No participants self-reported Inuit ancestry. Gender response options were man, woman, transgender man, transgender woman, or gender expansive

No participants identified as transgender or gender expansive (including people who identified as transgender men, transgender women, or gender non-confirming people)

### Findings of thematic analysis

From the data, we identified that participants made several considerations and adaptations in order to balance the perceived benefits and risks of their use of methamphetamine with opioids. Two overarching themes were identified to describe how participants adapted their ways of using for survival and wellness: (1) Personal safety behaviours, and (2) interpersonal safety behaviours. Personal safety behaviours encompassed behaviours that participants developed and applied to their own patterns of use that were sometimes influenced by others, but did not require other people in order to be performed. Interpersonal safety behaviours were behaviours that involved other people or services in order to be performed (See Table 2).

**Table 2:** Themes and behaviours

Theme	Behaviour
Personal behaviours for survival and wellness	Self-regulation via concurrent use, mode of use, and moderation Self-care (ensuring physical needs are met)
Interpersonal behaviours for survival and wellness	Using alongside peers^a^ Engaging with peer-led services Engaging with public health-led services

^a^Peer refers to friends, acquaintances, and other people who use drugs

### Personal behaviours for survival and wellness

Participants described personal safety behaviours they took when concurrently using methamphetamine with opioids in order to optimize benefits of use and reduce perceived risks. These were often adaptive coping mechanisms practiced in order to maintain a feeling of control over their use. Participants’ personal safety behaviours included efforts to self-regulate intake and to ensure that their physical needs were met before and during use (self-care).

#### Self-regulation

As the defining feature of our sample, all participants used methamphetamine with opioids. This was described repeatedly as a measure to achieve a sense of balance. Concurrent use of methamphetamine was described to provide a sense of stimulation while using opioids, and opioids create a sense of calming while using methamphetamine. Self-regulation refers to health behaviours described by participants to achieve and maintain a sense of balance in their personal health and wellbeing.

Some participants rationalized concurrent opioid and methamphetamine use as beneficial in terms of sensory experience, where methamphetamine use was seen to make the effect of the opioids last longer (saving money), and most importantly, concurrent use was thought to *reduce* risks of having an overdose. For example, one participant who reported regularly snorting methamphetamine and opioids described:

*To balance each other out, sort of like a yin and a yang, you know. Upper and a downer…... If you're doing some speed with your heroin, there's less chance you'll OD [overdose] on the heroin, so you're doing some speed with it as well, you know what I mean?* – David

Seeking the desired balance was sometimes reported to be difficult when using unregulated drugs. Participants described a sort of experimentation to achieve the right balance, trusting their own self-awareness as the cue to correct the dosage and feel safer.

For example, a participant who resided in a community in Northern BC described:

*Yeah, it usually depends-- the up first and then you use the other, you’ll feel the speed first, the meth first, and then just as that is dipping you’ll feel your down high with fentanyl. Sometimes you kind of use them to get one-- ‘cause if you do too much speed with meth, you get really anxiety and anxious. Sometimes you got to use a little more down just to gain that-- it’s like you’re trying to fight to be normal* - Jennifer

Some of the participants who injected drugs described mixing methamphetamine and opioids to reduce the frequency of administration and thereby the number of injections and associated risks of infection. For example, Jennifer described:

*Yeah, and it used to be because-- I used to do them separate. I kind of actually like that better. Because it’s getting hard for me to poke holes in it. It’s really hard for me to find a vein. So why I would to do that to myself twice, right?* - Jennifer

Participants reported various routes of administration for their methamphetamine and opioid use (smoking, injecting and snorting). Each had strong justifications for their choices that were often influenced by perceptions of safety. Many participants stated that they were making direct efforts to self-regulate their use to reduce risks of overdosing or over-damping. Choices were often based on personal experience, or observation of others, and although many participants considered all modes of use to have risk, they often justified their choice as what they perceived as safest for them. Participants who had witnessed overdoses or behavioural changes in their peers reported negative associations with the route of administration used by that peer and often consequently used this information to inform their own chosen route of administration.

Many participants had been at the scene of multiple overdose events and had made assumptions about risks of use associated with different routes of administration based on their observations. Some believed smoking to be a more dangerous route of administration for overdose risk, while others felt injecting was riskier. For example, David who reported regularly snorting methamphetamine described:

*I think injecting is the most dangerous because you can do too much easily. Most of the OD's (overdoses) I see is from shooting. I think it's potentially more dangerous than any other way because you're introducing god knows how much into your bloodstream so quickly.* - David

Route of administration was often an important aspect of the substance use experience in terms of enjoyment and safety. In particular, participants who reported using by injection often discussed the importance of safer technique in terms of both discretion and reducing health risks such as overdose and infection. For example, one woman who reported daily injection opioid and methamphetamine use stated the perceived risks of not practicing proper technique when using drugs by injection:

*If you know what you're doing and you've done it before, it shouldn't be taking that long.... cause if they miss, you can die, or you can get paralysed, all things -- all kinds of things can happen…. another thing is with methamphetamines, you have to be careful when you're injecting. It's a lot -- you can get -- if you miss, the methamphetamines is the stuff that will give you an abscess more than the down will.* -Tracey

Other participants reported pacing their use, using only small amounts at a time as a mechanism to control the risk of potential overdose:

*I don't do a lot. I'll do a little at a time and then If I need, I'll do a little more, you know. I never do a huge amount at one time 'cause that would be too dangerous for me. *- David

For some participants, perceived vulnerability to risk of harm was associated with reliability of the product. Many of the participants were confident that the drugs from their personal supplier were consistent. *“I'm confident in my – where I get my supply”* (Andrew), where there was doubt in the quality or a change in supplier, participants described using with extra caution. One participant who reported daily inhalation or smoking of methamphetamine and opioids stated:

*It's usually like I said, I don't just buy whatever, just off of anybody. I make sure it's somebody that I know, whatever, and I ask them if it's clean. I make sure to ask them when I'm purchasing. Is it clean? is it cut with anything? I don't just buy and use whatever, right. I'm really cautious with all this overdosing and shit going on, whatever. I have to be really cautious* - Nathen

#### Self-care

Nutrition, hydration, and adequate sleep were important aspects of self-care emphasized by participants in order to manage substance use. Where participants reported that their basic physical needs were not met, it was perceived that the vulnerability to overdose and other harms of illicit substance use increased. As methamphetamine and opioid were used concurrently by these participants, they described a range of challenging effects including lack of motivation to conduct daily tasks, decreased day-to-day functioning, and hypervigilance. Participants reported that the concurrent use of methamphetamine and opioids contributed to reduced desire to sleep or eat, but participants made special efforts to ensure self-care to reduce harms associated with their substance use. One participant spoke about the negative effects of sleep deprivation and stressed the importance of ensuring adequate sleep to protect their mental health:

*If you eat and you sleep every night, you’re okay. You can function and that’s what I do. I sleep every night and I also make sure I eat.* - Anita

Sometimes the inclusion of self-care into their daily routine was described as being something that had to be consciously self-enforced even when this was counter to their physical feelings. One participant who reported using methamphetamine and opioids daily and reported increasing their use at social events where they may stay up for multiple days, stated:

*I do make sure I eat. Even if I have to force myself, I still eat because I can't just go around, whatever, without any energy…. I'm like, yeah, well, fucking I'm not like you guys, I can't just fucking live solely off meth* – Nathen

For people experiencing homelessness and unemployment, ensuring access to basic needs, including food and sleep, was more challenging. Another participant who had experienced homelessness and was now housed in a private residence noted:

*Yeah, the longer you stay awake, the longer-- yeah, your body starts shutting down quite fast on you...Sleep and eat. That’s the difference is having a place and sleeping and eating.* – Anita

Access to these basic needs was also reported as challenged by inadequate and unreliable income. Lack of funds to support access to methamphetamine and opioid use often meant that participants’ supply of substances was irregular, which contributed to periods of increased vulnerability in terms of sleep deprivation and poor nutrition, often directly associated with the experience of withdrawal.

Participants each balanced the costs and benefits of their methamphetamine and opioid use alongside the cost of meeting their other basic needs. For some participants, balancing the cost of the substances along with the cost of their basic needs meant making decisions about which substance to prioritize. For example, one woman who reported daily methamphetamine use described sometimes having to make the decisions to prioritize her need for fentanyl, food, and a place to sleep over the use of methamphetamine:

*I have the power to say no, and-- when I have fentanyl in my system. Or when I just think back and-- where I just think back, think back in the clock-- what happened when you did this last time. You had no food in your system and you just did a shot of meth and-- yeah, no drug-- you had no fentanyl and no means to get any fentanyl or food or a place to sleep...*- Darlene

### Interpersonal behaviours for survival and wellness

Many participants noted the importance of community or peer support. While personal safety behaviours were enacted based on perceived vulnerability to risk and self-efficacy to control the risk, interpersonal safety behaviours appeared to be more complex. The supportive community must be found, feel safe, and accessible and be aligned with one’s personal needs so that agency is preserved. This could be in the form of a friendship group or services that are led by peers or public health professionals.

#### Using alongside peers

Participants in the present study reported either engaging with peers for support, or desiring peer support. The experiences of using alongside peers varied across the province. For example, participants who resided in more rural regions spoke of seeking communities where their drug use was more accepted and where the drug supply was more reliable. For example, one participant living in a small town in the north of the province described the challenges of using drugs in that community compared with larger urban centres in the south of the province.

*The difference is, is that there’s no help here. There’s no nothing. The drugs are bad. They’re more expensive* - Jennifer

For those participants who reported having peers who also used opioids and/or methamphetamine, there seemed to be a sense of safety in belonging to a group. This security was not only protective of health risks, but of not experiencing isolation in their routine of substance use. Some participants reported keeping a limited peer group, including only a small number of trusted contacts. For example, one participant who smoked methamphetamine and opioids daily reported sharing drugs and equipment with his close group of peers:

*My group of friends I use with are pretty small, right, so it's not like I'm sharing with everybody who walks up or anything* – James

When a peer group was not available, or behavioural changes and vulnerability to stigma were perceived as being more serious or concerning than the risk of overdose, participants described needing to hide their drug use. Concealing use by using alone was described as a safety behaviour that helped to mitigate risk. For example, one participant reported not wanting to be in the community or with others when he used due to the effects his substance use had on him:

*If I've used too much like, I become flaily… and sometimes I just -- it becomes very apparent which means I'm not very, like, I don't like to be out in the community very much* - Andrew

where overdose was perceived as the more salient risk, however, using alone was considered a high-risk behaviour. The phrase, “don’t use alone”, was also a common sentiment shared by participants. It must be noted, however, that according to some participants, in order to use with others, one must feel safe doing so. This following quote illustrates how one participant described how people who use drugs sometimes have to weigh the risks of stigma associated with admitting drug use to others with the risks of overdosing. In the following quote, Jennifer, a peer advocate working to encourage others not to conceal their own drug use, explains how people who use drugs sometimes have to weigh the risks of stigma associated with admitting drug use to others with the risks of overdosing.

*You don’t want anyone to find out you’re using because they look down on you so much because of lack of knowledge and lack of education. People don’t understand. They think that, they don’t understand what opiate addiction is. They don’t understand that this person can’t get up and go to work normally without using the opiate. So they think that it’s a choice. So because of the stigma, people hide it. And I have a thing, that if you hide it, you die. Because if you don’t-- if you’re in the bathroom hiding it and you don’t tell your family you’re using it, nobody’s going to check on you. Nobody’s going to make sure you’re okay. If they hear a bump in your bedroom they’re not going to come look.* - Jennifer

#### Engaging with peer-led services

A number of peer-led harm reduction services (i.e. supervised consumption sites, overdose prevention sites, community outreach organizations) are available in communities across BC. Some of the participants in the present study were employed by peer-led services. This provided purposeful work and opportunities for community engagement. This engagement allowed participants to reduce risks for one another, but also supported participants to self-regulate their methamphetamine/opioid use. For example, one woman who was employed providing peer-led harm reduction services explained the meaning she found in her work, and the subsequent reductions in her own use:

*I think when I was really depressed and before I got this job doing—helping people, I started using a lot more. But now that I feel better about myself I definitely use less.* -Jennifer

Participants outlined a strong sense of solidarity and responsibility for mutual protection and provided tools to help one another seemed to reduce the risk of harm. Having naloxone and knowing how to use it was spoken of as a basic and fundamental responsibility among participants. For example, one man outlined accessing naloxone not only for his own protection, but also to protect his peers:

*I always make sure, like, I got my kits, the Narcan kits and everything. I got 6 of them in my bag all the time. Because it's not just me. It's, like, all my brothers and sisters. And I always make sure everybody's ok…. I got all my first aid (certifications).* - Michael

Many of the participants noted that because illicit drugs are unregulated, the content or potency of each sample is unreliable. Some participants described placing confidence in their “dealer”, trusting them, as their peers, to vouch for their own supply. Indeed, one participant who sold drugs stated that it was *“part of my ethical thing”* to try the product before selling it to *“make sure it’s not going to harm anybody”* -Oliver.

In this way, in the context of our study, peer support was described as extending beyond the immediate group of peers to a sense of responsibility to and trust in other connections within the wider community. One participant further explained that he would not sell a product that he would not use himself and would only purchase a supply from his own trusted peers:

*I don’t use as much as I used to, but I still use. And I don’t buy unless I know somebody that – unless it’s somebody I know. I used to sell it myself, whatever, and I wouldn’t sell what I wouldn’t smoke myself, right* - Nathen

#### Engaging with public health-led services

In addition to discussions of the behaviours they practice individually, or with the support of peers, there was some discussion among participants about engagement with services provided by public health (e.g. overdose prevention services, drug checking services, etc.). In many cases, participants shared a hesitancy to engage with these services, and there were a wide range of reported experiences with such services.

Those who were aware of, and comfortable using, drug checking facilities reported positive experiences with these services. For example, one woman who reported daily injection of both opioids and methamphetamine reported appreciating having access to services that would tell her exactly what substances were detected in her samples:

*Yeah they have drug testing at all these injection sites at different times. And you know what. If the one doesn't have it, the other one will. It really does help … they actually have the test where it will tell you specifically what is exactly in the damn things instead of just saying yes for one thing, you know what I mean? So it's better to go to those* -Tracey

Participants reported accessing harm reduction sites such as supervised consumption sites (SCS) and overdose prevention services (OPS) to reduce risk of harm. Nevertheless, many participants noted limitation in these services, for example, a lack of services for people who do not inject their drugs (i.e. who use by other routes of administration such as smoking). In some cases, participants reported adapting their modes of use to be able to access the services given the risk of harm if using alone (and not accessing the service) was so high. For example, one participant reported:

*I know people who started injecting just because of that and that’s bad… now they inject because they can be with [the service] yeah, so they can do it safely. Because nobody wants to die. And it’s scary. It is ‘cause it could happen to anybody. And not much you can do about it. There really isn’t many steps you can take on your own to make it safe. The only steps we can take are finding someone to be with you.* - Jennifer

Other services include pharmacotherapies and detoxification programmes. Reported experiences of these services were mixed. For example, one participant outlined the desire to access detox and then treatment, but that the timing of these services being available never seemed to line up for her:

*It would be beneficial if you could get it all lined up so that you could go to detox, stabilization and then out to treatment. But I’m pretty sure, like, fifty percent of the time that that doesn’t happen.* – Carla

Another participant reported an overall lack of services in their rural community, whereby the only form of support available to people who use drugs was the psychiatric ward at the local hospital:

*It is. There’s no support. The only place you can go here—if you decide you want to stop doing drugs in the middle of the night or even during the day and you go for help, the only place you can go is the psych ward. And who—I’m not going to the psych ward. Like, who goes to the psych ward by choice to get off drugs?* - Jennifer

Some participants reported that in cases where they were able to access services they experienced stigmatization from health care providers. Those who had a positive experience of services tended to stress the importance of being heard and validated for their individual experience and being provided with flexible treatment options that aligned with their goals. Participants expressed that feeling safe and supported through engagement with any service is paramount to achieving positive outcomes:

One man who sought out access to opioid agonist treatment reported being heard and cared for by the health care providers he was engaged with and that this helped him achieve progress:

*Yeah, it was a big decision to just even put yourself into the hands of the healthcare professionals, but they really did take care of me and really did hear me. And I came with the right reasons, and I got -- I made some super progress* – Robert

While some participants described their attempts at accessing harm reduction and treatment services for both their opioid and stimulant use, in many cases, the need for intervention or support relating to reducing illicit stimulant use, specifically, was lacking. For example, one participant described:

*The doctor here said we usually see people’s meth use go down when they’re on, like, when they finally get off fentanyl or whatever and stuff and they’re using methadone. They said they usually see the meth use go down but the doctor I was seeing before for Suboxone didn’t really say anything else about it. She was just, like, oh, so you’re still using meth? And I was, like, yeah. And she’s, like, okay. And that was that.-* Fiona

Some participants described having access to prescribe pharmaceutical alternatives to the illicit drug supply as helping to reduce their illicit substance use. Experiences with these medications were mixed and prescribed alternatives of opioids and stimulants were reported as not always being available or meeting participants’ needs. For example, one participant described preferring to smoke stimulants, and therefore, not finding the currently available stimulant to be a suitable option for him:

*Like even to date, the only reason I still smoke [methamphetamine] is because I enjoy it. I could get, I can't remember what It's called, but it's like Dexedrine or something…. There's actually a pharmaceutical replacement, but I don't want it. I want to smoke meth.* -Wesley

Other participants described a preference for a prescribed rather than illicit stimulant, based on the view that it was safer than methamphetamine. For example, one participant described:

*It’s a lot, like, you feel a lot nicer with it [Dexedrine] than you do with, like, even just the difference of that and meth.... It’s like it doesn’t hang around in your system as long, it feels like. Like you don’t have as much toxins obviously, right.* -Adam

One woman who had attention deficit hyperactivity disorder (ADHD) symptoms described the benefits she experienced when accessing a prescribed stimulant (using a family member’s prescription). However, she was not able to secure a prescription for herself:

*So I took 3 days’ worth of [Dexedrine] -- I got back taxes done, my house was clean, I was on the level. My life was going good, so I told the doctor that and I asked for another prescription and they said no. -* Paula

## Discussion

Our study found that people who use methamphetamine with opioids adapted their patterns of use for their own survival and wellness and for that of their peers. This included personal behaviours to modify their use via self-regulation and self-care strategies and utilize interpersonal behaviours, including relying on peers and service providers to promote survival and wellness. The beliefs that supported participants’ behaviours were generated through direct experience or from observation of peers. The focus on survival and wellness as responses to risk among participants of our study is aligned well with recent studies that have emphasized the importance of shifting away from a focus on “risks”. Likened to the responses of participants in our study, this literature focuses on the resilience practiced in the daily lives of people who use drugs [15], whereby people become active agents of harm reduction for themselves and their communities [16].

Risk of harm from illicit substance use is produced through interactions between individuals and the physical, social, economic, and policy environments in which they live [17]. People who use drugs make choices around their use to manage these risks. For example, perceived risks of illicit substance use have, in prior studies, been shown to impact choice of drug(s), dosages, motivations to use, and modes of use; peer supports and improvements in self-efficacy have been found to be the main areas of focus for strategies to reduce potential risks [18]. Some misconceptions [6] about concurrent use of methamphetamine with opioids were noted. For example, some participants believed that using methamphetamine with opioids could protect them from opioid overdose, while others justified their preferred mode of use (i.e. route of administration) based on perceptions of safety. In the present study, more participants reported smoking methamphetamine or opioids (45.5%) than injecting (40.9%). This was consistent with the overall population included in the 2019 HRCS where more than 60% of participants reported smoking heroin or fentanyl in the prior 3 days [19]. Traditionally, smoking has been promoted as safer than injecting due the reduced risk of blood-borne infection [20, 21]. Recent data from the BC Coroners Service, however, show an increase in illicit drug toxicity deaths where smoking was identified as the route of administration (from 28% of deaths in 2017 to 40% in 2019) [22]. More research is required to consider the various nuances of different modes of use and associated risks in the context of an illicit unregulated drug supply. It is the responsibility of public health practitioners, researchers, and advocates to ensure these findings are made widely available through educational campaigns so that people who use drugs can be supported to make informed decisions about their substance use, including route of administration.

People who concurrently use methamphetamine and opioids appear to be a growing population in BC [7] and among people who use drugs more broadly in North America. It is important that the unique risks of concurrent methamphetamine and opioid use, especially in the context of an unregulated illicit drug supply, be well communicated to people who use these substances. Furthermore, strategies for wellness and survival in this context must also be widely shared. In our study, many participants reported relying on peers and peer-led services for support and education. Prior studies of health behaviour change have demonstrated the effectiveness of peer-supported interventions in promoting change [23]. As such, in BC, there is an important role for peers with lived experience of methamphetamine and opioid use to lead education efforts and to promote wellness within this population. Such education could be delivered through pre-existing peer-run harm reduction services, which have been shown to promote service engagement and improve health and social outcomes [24].

In our study, there was a strong reliance among most participants on peers to monitor use. Participants also often reported relying on their source of drugs, noting that having a trusting relationship with the person selling them their supply was an important behaviour for safety.

While it is important to highlight the risk of harm that persists within an unregulated supply, trusting relationships can serve as a means of information-sharing and social support. Nevertheless, the contents of the supply often remain unknown even to the person selling them [25]. In BC, the illicit drug supply is associated with increasing numbers of drug toxicity events and deaths, and people who use both methamphetamine and opioids face compounded risk of drug toxicity [26]. While the reliance on peers to communicate with one another, and to supervise one another’s use represents an important behaviour practiced to support survival and wellness, given the volatility of the drug supply in BC, safety and survival remain a challenge. As such, there are increasing calls in the province to scale up harm reduction and treatment interventions for those interested in accessing them [27, 28].

Harm reduction and treatment interventions for people who use methamphetamine with opioids were discussed by participants in the sub-theme “*engaging with public health services*”. There were a number of examples of difficulties with accessing or engaging with these services as expressed by participants. For example, more services were reported to be available for those who injected methamphetamine or opioids, leaving those who used via other routes (i.e. smoking) underserviced. Furthermore, participants in the present study were currently engaging in both methamphetamine and opioid use together, and services for concurrent use were lacking. While some participants discussed desires for increased access to treatment (e.g. opioid agonist treatment), some participants’ narratives were not centred around goals of abstinence. Instead, many discussions focused on practicing behaviours that allowed them to survive and to seek wellness while they maintained the use of both methamphetamine and opioids. In the absence of abstinence as a goal, public health interventions must be centred on providing supports that help people stay safe.

As identified in prior studies of people who concurrently use stimulants and opioids [29–31], our study sample had diverse patterns of opioid and methamphetamine use, including varying frequencies and routes of administration. Across this diversity, the sample consistently practiced a range of behaviours to promote their own survival and wellness, and that of their peers. In BC, Canada, and North America, there are increasing calls for the provision of a safer supply of drugs, including both opioids and methamphetamine [32–34]. Greater access to safe drugs would reduce the overwhelming burden placed on people who access the illicit supply of drugs to consistently practice safer behaviours that reduce the potential risks of harm. Interventions seeking to further support people who use methamphetamine and opioids, not just in fostering survival and wellness but also in promoting safety, must be adaptable and diversified in order to respond to the various preferences of drug types and routes of administration [35] reflected among this population.

There are a number of limitations of our study to consider. Despite having recruited participants from across the province, with various levels of engagement in harm reduction services, who engage in various frequencies, modes of administration of opioids and methamphetamine participants might not be representative of all people who use methamphetamine with opioids in BC. Nevertheless, participants represented all geographic regions of the province, and participants also reflected a diversity of historical and current patterns and routes of opioid and methamphetamine use (e.g. smoking, snorting, injecting, etc.).

Furthermore, while the study included a mix of people who identified as men, and as women, no participants identified as transgender men, transgender women, or gender non-conforming. Future research studies could be focused on understanding the unique methamphetamine and opioid use behaviours and strategies for survival and wellness practiced in this population. One half of participants self-reported Indigenous ancestry, including First Nations and Métis. As services are expanded to meet the harm reduction and treatment needs of people who concurrently use methamphetamine and opioids, it will be critical for researchers, clinicians, and decision-makers to work with a diversity of Indigenous peoples and communities to create services that are equitable and accessible and culturally safe for Indigenous people who use drugs [36, 37].

In studies of self-reported substance use, stigma and social desirability bias might limit the degree of openness to which participants are willing to discuss their personal substance use. While this is possible within the context of our study, we have attempted to minimize these potential sources of bias by having interviews conducted by peer researcher assistants (PRAs) and researchers who were able to connect with the participants and engage in open and trusted conversation that allowed for the collection of rich data. Furthermore, engagement with the PEEP consultation and advisory committee helped to reframe the focus away from safety and risk, towards survival and wellness. This refocus not only reduces the potential stigmatization of drug use as necessarily risky, but also allows for an account of the skilful ability and expertise held by people who use methamphetamine and opioids to actively adapt their behaviours to promote survival and wellness for themselves and their communities. This involvement helped to ensure that the research question and analysis were reflective of the lived experiences of people who use drugs.

## Conclusion

This manuscript identified diversity in participants’ methamphetamine and opioid use (i.e. frequency, route of administration), and a subsequent range of behaviours that were performed to improve wellness and survival while using methamphetamine and opioids. Some of participants’ behaviours were practiced individually, while others relied on a community of peer support, or public health service provision. Nevertheless, participants identified many gaps in available services to meet their diverse needs. In the context of an unregulated illicit drug supply in BC that is associated with increasing toxicity-related events and deaths, harm reduction and treatment responses must be robust and adaptable to respond to the diversity of patterns of substance use among people who use opioids and methamphetamine concurrently, so as to not perpetuate harm and leave people behind.

## Data Availability

Data sharing is not possible at this time given we do not have ethical approval from our REB, nor consent from study participants to share transcripts.

## Abbreviations

CUT Meth OD: Concurrent Use and Transition to Methamphetamine among persons at risk of Overdose
OAT: Opioid agonist treatment
PEEP: Professionals for Ethical Engagement of Peers
PRA: Peer research assistant
PWLLE: People with lived and living experience of substance use
PWUD: People who use drugs
